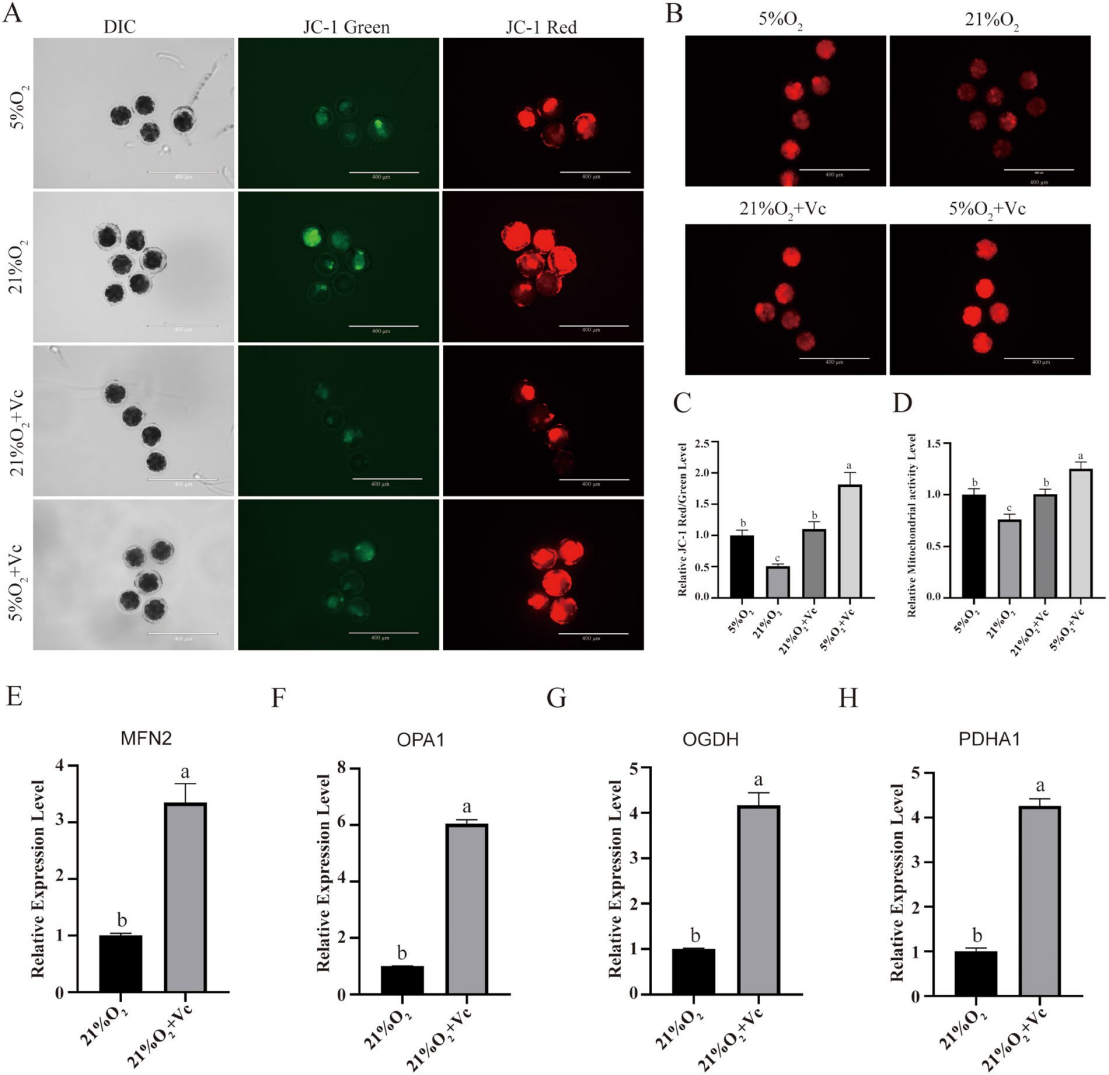# Correction

**DOI:** 10.1080/10495398.2025.2595390

**Published:** 2025-11-27

**Article title:** Vitamin C enhances the *in vitro* development of early porcine embryos by improving mitochondrial function

**Authors:** Lei Wang, Liu She, Peng Qiu, Meiyun Lv, Yunchuan Zhang, Yunjia Qi, Qin Han, Deshun Shi and Chan Luo

**Journal:** *Animal Biotechnology*

**Bibliometrics:** Volume 35, Number 01, pages 01 - 14

**DOI:** https://doi.org/10.1080/10495398.2024.2404043

When the article was first published online, Figure 5A, the image of JC-1 Red in the group of 21% (Line 2) didn’t match other images. Now this has been updated with the revised figure provided below and republished online.